# Antibacterial and Anti-Inflammatory Potential of *Morus alba *Stem Extract

**DOI:** 10.2174/1874210601812010388

**Published:** 2018-05-23

**Authors:** Ichaya Yiemwattana, Niratcha Chaisomboon, Kusuma Jamdee

**Affiliations:** 1 Department of Preventive Dentistry, Faculty of Dentistry, Naresuan University, Phitsanulok, Thailand; 2 Research Laboratory, Faculty of Dentistry, Naresuan University, Phitsanulok, Thailand

The correct a new genus name of bacteria is provided and replaced online in whole article which is mentioned as under:

*Aggregatibacter*

The published genus name of bacteria was:

*Actinobacillus*

